# Role of trefoil factors in maintaining gut health in food animals

**DOI:** 10.3389/fvets.2024.1434509

**Published:** 2024-11-19

**Authors:** Yewande O. Fasina, Temitayo O. Obanla, Deji A. Ekunseitan, George Dosu, Joseph Richardson, Oluwabunmi O. Apalowo

**Affiliations:** Department of Animal Sciences, North Carolina Agricultural and Technical State University, Greensboro, NC, United States

**Keywords:** trefoil factor family, peptides, gastrointestinal mucosal injury, mucins, epithelial repair

## Abstract

It is imperative to preserve the integrity of the gastrointestinal system in spite of the persistent existence of harmful chemicals and microbial flora in the gut. This is made possible by essential healing initiators called Trefoil factors which helps in mucosal reconstitution and tissue development on the gastrointestinal surface. The trefoil factors are a class of abundant secreted proteins that are essential for epithelial continuity (TFFs). Trefoil factor family (TFF) proteins are biologically active peptides that play significant role in safeguarding, restoring and continuity of the gastrointestinal tract (GIT) epithelium, through collaborative modulations with mucins in the mucosal layer. These peptides are readily produced in reaction to epithelial damage in the digestive tract, thereby contributing to the healing and restituting of the epithelial layers of the intestine. In addition, considerable evidence indicated that TFF peptides trigger proliferation, migration and angiogenesis, all which are crucial processes for wound healing. There is also increasing evidence that TFF peptides modulate the mucosal immune system. These protective properties, suggest that dietary manipulation strategies targeted at enhancing the expression and synthesis of TFF peptides at optimal levels in the GIT epithelium, may constitute a plausible alternative strategy to the use of in-feed antibiotic growth promoters to maintain epithelial integrity and promote resistance to enteric pathogens. This review describes TFF peptides, with importance to their biological functions and involvement in gastrointestinal mucosal protection and repair in food animals.

## 1 Introduction

The gastrointestinal tract is a complex environment that assembles myriad of components such as peptides, cellular matters, pathogens, microorganisms and nutritional biomaterials. The presence and interaction of these components determines the outcome put up against threat or challenge to the system (1). The GIT harbors immune cells and molecules that grants immunity and biological responses to pathogens and toxins made possible to protect the lubricated epithelial surface of the GIT. Consistent with these functions, it's interesting to note that TFFs are mostly expressed in the healthy gastrointestinal (GI) tract (2), thus it makes sense that they would either positively or negatively correlate with stomach disorders. The peptides known as the trefoil factor family (TFF1, TFF2, and TFF3) are essential for the upkeep, preservation, and repair of the gastrointestinal system.

Intestinal trefoil factor (ITF) is an important member of TFF domain peptides with pivotal roles in the protection of the intestinal epithelium (1, 3, 4). There are three secretory proteins (12-22KD) that make up TFF, and are often produced in adequate quantity in the tissues containing cells that secret mucus (3). TFF proteins are readily produced in reaction to epithelial damage in the digestive tract (1, 4) thereby contributing to the healing of the gastrointestinal epithelium (5). When an injury occurs to the epithelium, TFF are produced close to the injury site and migrate to the site by a process that causes a rapid change in the shape and volume of the cells, thereby allowing free flow of water in and out of the cells (6). There are three known mammalian Trefoil Factors namely TFF1 or the pS2 (7), pancreatic spasmolytic polypeptide TFF2 (8), and TFF3 (or ITF) (1, 9). The distribution of TFF cut across numerous tissues such as Liver, Pancreas, Kidney, brain salivary gland as well as the respiratory tract (10). The isomers of TFF are found in the gastrointestinal tracts with variation in distributions (10). The TFF1 is a 9-KDa mucin-associated secretory protein primarily expressed in the gastrointestinal (GI) tract mucosa, and is characterized by a 38- to 39-amino-acid trefoil domain which contain six cysteine residues that form a cloverleaf disulfide structure (11). This structure renders TFF1 resistant to both acid and enzymatic breakdown, and thus perhaps responsible for the ability of TFF1 to inhibit tumor growth in the gastrointestinal tract (GIT) (12). TFF1 exerts its protective action by stimulating the restitution of the tissues destroyed as a result of inflammation and ulceration (12). Pancreatic spasmolytic polypeptide TFF2 is secreted by the antral and pyloric region of the stomach, and the Brunner's glands of the duodenum (13). In a healthy animal, there is an interaction between TFF2 and mucins that often lead to development and maintenance of the mucus barrier in the gastric and duodenal sites (14). At the site of an injury, TFF2 can be rapidly expressed in the epithelia of the entire GIT and critically contribute to epithelial “restitution” and regenerative processes (9). Furthermore, TFF could modulate immune responses, cell chemotaxis, and cytokine release (15). The gastrointestinal mucosa is the primary site for TFF3 secretion (16). From this site, TFF3 is excreted from the granules with mucins onto the surface of the epithelium, thereby forming a protective mucosal layer. The mechanism of action of TFF3 is such that it stimulates the activation of extracellular signal-regulated kinase/mitogen-activated protein kinase and activates serine phosphorylation of Akt, a kinase associated with apoptotic pathways (17, 18), thereby modulating the E-cadherin/catenin cell adhesion complex in various ways. For instance, TFF3 peptide caused reduction in the level of E-cadherin, β-catenin, α-catenin and the adenomatous polyposis coli (APC) protein in HT-29 cells in, consequently resulting in significant alterations in cell aggregation, detachment from the substratum, and translocation of APC from the cytoplasm to nucleus (19).

Since TFFs are mostly expressed in the healthy gastrointestinal (GI) tract, there exist correlation, either positive or negative, between them and stomach disorders (20). This review aims to examine the significance and therapeutic potentials of TFF in the gastrointestinal tract health, provide current knowledge on the target factors and steer future research toward their perceived mechanisms and clinical applications in gastrointestinal disease management.

### 1.1 Trefoil factor (ITF): types, structure, and functions

The process of breaking down and absorbing consumed food and liquids is carried out by the gastrointestinal (GI) system. The intricacy of the GI tracts and complex nature of biological processes that occur with the system, preserving the gastrointestinal tract's integrity and homeostasis is crucial even in the face of constant exposure to microbial flora and other detrimental agents. The gut tissues and epithelium are designed to express polypeptide growth factors capable of stimulating cellular growth, proliferation, or differentiation after attachment to surface membrane receptors (21). These factors include epidermal growth factor (EGF), transforming growth factor beta (TGF-β), insulin-like growth factor (IGF), hepatocyte growth factor (HGF), fibroblast growth factor (FGF), trefoil factor, wingless (Wnt) family, and Hedgehog (Hh) family proteins (22). The latter three has been identified over the past few decades and discovered to play mitogenic roles in the development and adult function of the GI tract in animals. TFF peptide is discovered in both serum and luminal fluid of the GIT. TFF has been reported to be expressed in a cell-specific manner on the mucosal surfaces of normal gastrointestinal tissues (21). ITF proteins have been demonstrated to have important functions after mucosal damage by improving superficial cell resistance, lowering epithelial cell permeability, strengthening the cell-to-cell connection in intestinal mucosal injury, suppressing apoptosis and inflammatory signaling (2).

**Figure 1:**
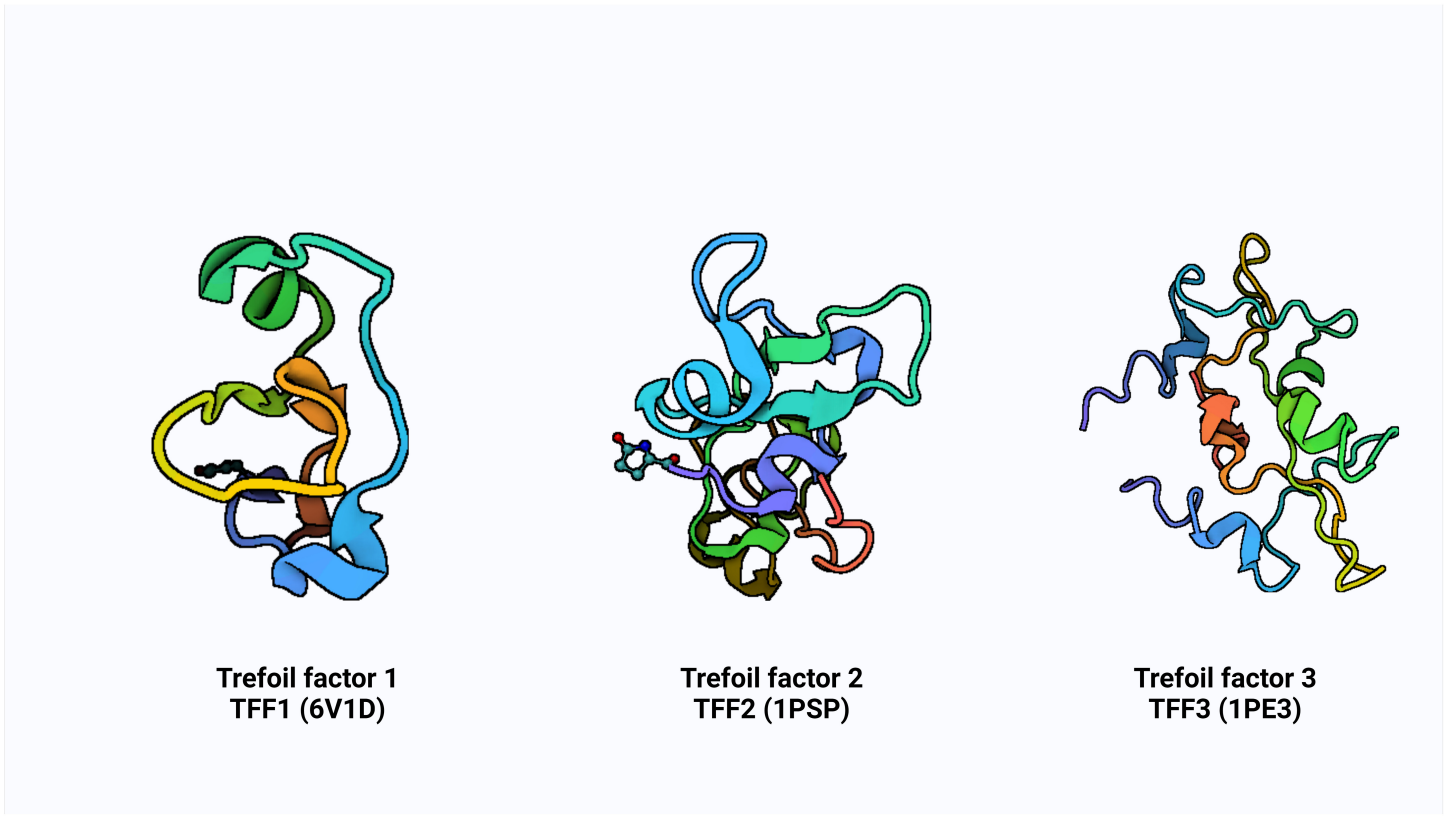
The PDB structure of TFF1, TFF2, and TFF3. Created with Biorender.com.

Additionally, some research has shown that ITF reduces the inflammatory response by preventing the digestive tract's production of pro-inflammatory factors (23, 24). These small molecular peptides factors called TFF are produced by the goblet cells scattered across the absorptive cells onto the gastrointestinal tract's surface. It is believed that mechanism of action of TFF is via luminal secretion of the peptide from the epithelial cells lining the GI tract, suggestive that systemic TFF peptides can be secreted into the gastric lumen in tandem with mucus secretion from mucous cells (10). The concept of growth factor started in the 80′s with the identification of spasmolytic protein SP (25) and pS2 (7). The advancement in knowledge and efforts over subsequent decades in the identification of some cell division activities, proteins classified trefoil (TFF) and subsequent of a third ITF in mammals (9). The pS2, spasmolytic peptide, and intestinal trefoil factor which share a distinct motif of six cysteine residues defined as “trefoil” domain, were renamed with the current nomenclature of TFF1, TFF2 and TFF3, respectively, in 1997 at the Philippe Laudat Conference (26, 27). The protein data bank (PDB) structure of TFF1, TFF2, and TFF3 is shown in Figure 1.

The TFFs are small secretory peptides with three loop structures that contain a highly conserved motif of cysteine disulfide bonds that maintain the functional stability of the protein. Depending on anatomical location, TFF proteins are both constitutively and inducible expressed (28). The trefoil, or P domain, is a conserved 38–39 amino acid motif containing six cysteine residues held together by three pairs of disulfide bonds configured 1–5, 2–4, and 3–6 (28, 29). It is a collection of secreted peptides with a C-terminal dimerization domain and a trefoil domain (s). The TFF2 has two trefoil motifs as a result of chromosomal duplication, whereas TFF1 and TFF3 only have a single trefoil domain (9, 30, 31).

TFF1 is a monomer that consists of a single trefoil domain, which includes about 40 amino acids forming a stable three-loop structure. This domain is characterized by six conserved cysteine residues that form three disulfide bonds, creating a compact and stable structure (26, 29). TFF1 is glycosylated and this influences its protective function in mucosal tissues. TFF2 is a dimer, composed of two trefoil domains, making it structurally different from TFF1 (Figure 1). These domains are also stabilized by three disulfide bonds in each domain and can form dimers through non-covalent interactions, and this dimerization is critical for its biological activity (26) and it is also similarly glycosylated to TFF1. TFF3 is a dimer, much like TFF2, but can also exist in a monomeric form. It contains a single trefoil domain, similar to TFF1, but typically forms dimers through disulfide bonds between two TFF3 monomers (26). The dimerization of TFF3 enhances its mucosal protective properties and like TFF1 and TFF2, TFF3 is also glycosylated.

The cell-specific patterns of occurrence of TFF expression result in a regional colocalization of specific TFF proteins and secretory mucins, providing a potential molecular basis for the functional mucosa specificity. Trefoil factor family peptides are typically co-secreted together with mucins and interact with mucins in the lumen to enhance protective barriers through mucosal innate immune defense, mucosal repair, and prevention of the infiltration of microorganisms and toxin insults (32, 33). TFF proteins and secretory mucins colocalize in certain regions as a result of cell-specific TFF expression patterns, which may provide a mechanistic explanation for the functional mucosa specificity. It is significant to note that expression patterns of TFF proteins vary greatly among infection specific, species and age-related. Early in the development of the embryo, TFF expression takes place, with the gastrointestinal tract expressing all three TFF proteins in unison with individual expressions becoming more restricted resulting in cell-specific patterns in adult (34, 35).

The expression of TFF is modulated by different factors. Expression of TFFs in the gastrointestinal tract is abundant and is second, in weight of protein, only to the mucins. TFF1 is mostly restricted to the pit cells of the stomach, TFF2 to mucous neck cells of the gastric gland, and TFF3 to goblet cells of the small and large intestine (22).

#### 1.1.1 Interactions and regulations with other immune cells

**Figure 2:**
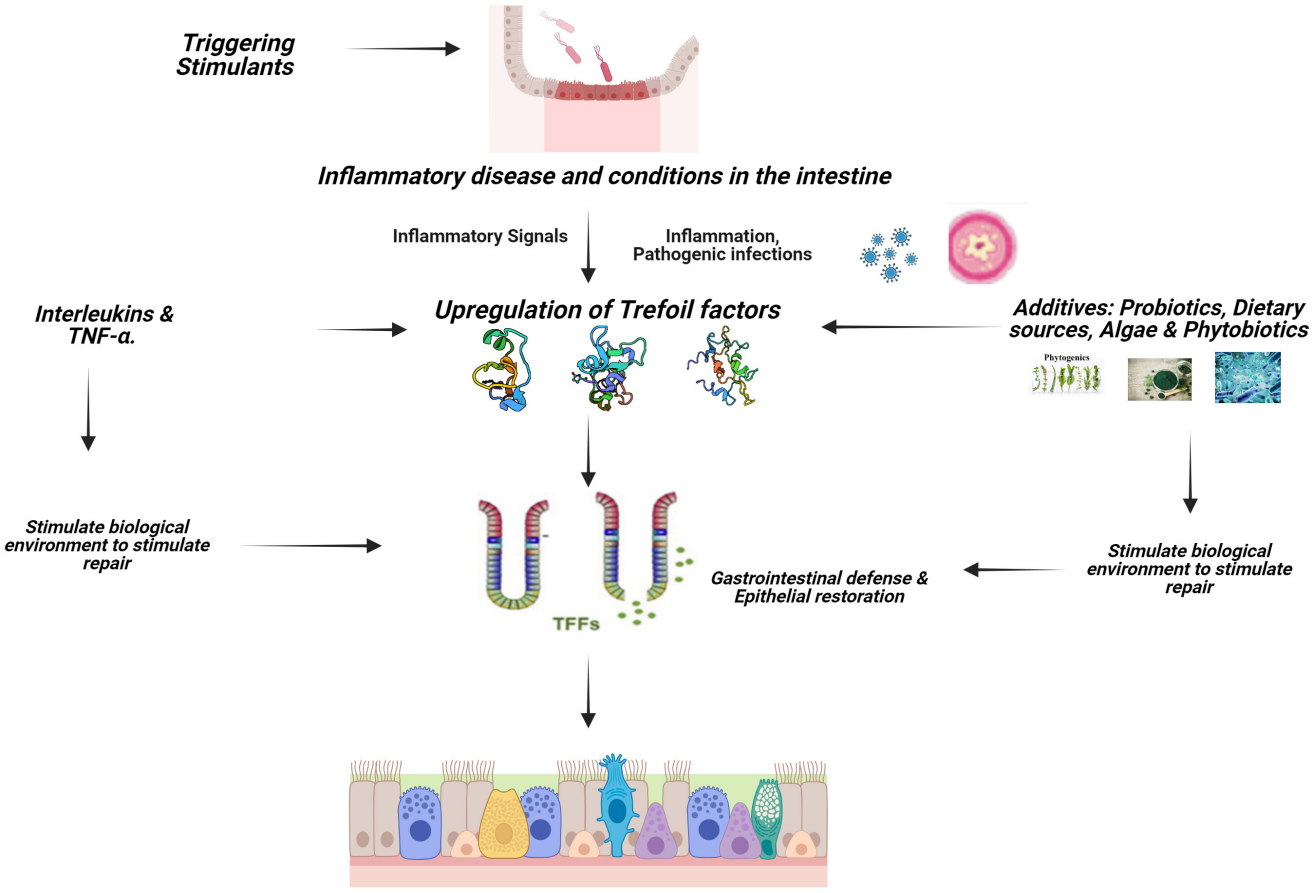
Signaling mechanism of TFFs in animals. Created with Biorender.com.

The immune system of the mucosal of the GIT is crucial because it keeps pathogens from gaining entrance into the body system while simultaneously facilitating the transfer of nutrients from the intestinal lumen to the systemic circulation for proper function (1). TFF peptides are major secretory products of mucous epithelia and play a multifunctional role in cytoprotection, apoptosis, and immune response. TFF influence the activity of immune cells and cytokines, helping to regulate inflammation and maintain immune homeostasis in the gut (Figure 2). Trefoil factors play significant role in the homeostasis of the gastrointestinal tract and are expressed rapidly in response to injury and are up-regulated in inflammation and ulceration (3, 21, 22). This perceived protective is achieved through regulations by pro-inflammatory and anti-inflammatory cytokine expressions (32). Cytokines such as IL-1β and IL-6 down-regulate TFF genes expression by transcriptional repression in GI cells; IL-6 represses TFF1, IL-1β and IL-6 inhibit TFF2 and TFF3 transcription, TNF-α (acting via NFκB transcription factor) decreases trefoil expression during inflammation thereby delaying mucosal restoration, and cytoprotection (36) while IL-4 and IL-13 induce TFF2 (37).

Pattern recognition Toll-like receptors (TLRs) is a key player in the initiation of an inflammatory process, TLR2 regulates TFF3 expression in the gut to control terminal GC differentiation, which protects the intestinal mucosa against apoptosis (38). The gut epithelial activating transcription factor 4 (ATF4) plays an important role, as its deficiency results in decreased peptide trefoil factor 3 levels (39). The identification of TFF-interacting proteins presents a potential regulatory mechanism for TFF function in the GI tract.

### 1.2 Trefoil factor family peptides (TFF1, TFF2, and TFF3)

#### 1.2.1 Trefoil factor 1

TFF1 is mainly expressed in the fundus and antrum of gastric mucous cells but with low expressions in ileum, colon, salivary glands, pancreas and respiratory tract (2). The major site of expression of TFF1 is primarily in the gastric foveolar cells and surface epithelial cells across the entire stomach, and it is also present in the upper ducts of brunner's glands localized in the proximal duodenum, salivary glands and gastric juice (10). TFF1 plays an important role in the number of differentiated cells in the epithelium of the stomach. TFF1 is induced by hypoxia-inducible factor-1 (HIF-1α) pathway resulting in the inhibition of activation of IL6-STAT3 pro-inflammatory signaling axis that stimulate gastric lesions (40). The Tff1 and the gastrokine genes (Gkn1, Gkn2, and Gkn3) are expressed selectively in the duodenum and but at much lower levels when compared with the stomach (41). The subcutaneous administration of TFF1 has been recorded to suppress tumor growth *in vivo*, affirming that TFF1 enters the bloodstream to reach tumor cells, where it functions as a tumor suppressor.

TFF1 help cells to counteract bacteria colonization and the development of a chronic inflammation. TFF 1 induces the following function with respective interactions; MUC2/MUC5AC (protection of the mucosal) (42), Fragment of IgG Binding Protein (FCGBP) (binding of microorganisms) (43) and Gastrokine 2 (GKN2) (antiproliferative and pro-apoptotic and homeostasis) (44).

#### 1.2.2 Trefoil factor 2

The spatial localization of TFF2 in the gastrointestinal tract varies among different species and this offers highlights on species-specific expression patterns and the potential functional diversity of TFF genes across species (13). TFF2 peptides expressions have been found in acinar cells of the pancreas, gastric, pyloric and Brunner's glands (Humans and rodents), mucous cells of stomach and throughout the intestine (13, 18). The functional diversity can be increased by the interactions of each trefoil loops with different substrates such as the amino acids. Expressions shift suggests a spatial transition of expression abundance from upper intestine to lower intestine as the gut matures as illustrated in the study in chickens (45). It has been particularly observed that TFF2, when added to a mucin solution, greatly improves the viscosity and flexibility of the mucus, indicating that ITF plays a vital role in repair responses to maintain mucosal surface integrity during pathological processes (46). Trefoil factor-2 is a stable secretory protein expressed in gastrointestinal mucosa responsible for protecting the epithelial layer from insults, stabilizing the mucus layer and promoting healing of the epithelium Two trefoil motifs identified and present in TFF2 is believed to be essential for the protein's proper function as *in vitro* recombinants with a single trefoil domain was discovered to lose its anti-apoptotic properties but continue to stimulate cell migration (45). TFF2 and Fragment of IgG Binding Protein (FCGBP) interaction has been proven to participate in intestinal immunity (mucosal protection). TFF2 binds to the mucin MUC6 thereby stabilizing the inner insoluble gastric mucus barrier layer (47). The interaction of TFF2 and MUC6 also alters the viscoelastic properties of gastric mucous gels thereby contributing to the gastric mucosal innate immune defense.

#### 1.2.3 Trefoil factor 3

Trefoil factor family protein 3 (Tff3) is a small peptide (59 amino acids; 7 kDa) that is a member of the trefoil factor family proteins (Tffs) (48), and it is specifically distributed in the surface of the intestinal mucosa (49). It is known as intestinal trefoil factor and was identified from a rat cDNA sequence in 1991 (50, 51). It is a stable secretory protein expressed in gastrointestinal mucosa, selectively dispersed on the intestinal mucosa's surface (49) and has been discovered to be predominantly expressed by goblet cells of the large and small intestines (50, 52), and also interact with mucins on the apical cell surface (10). TFF3 plays a significant role in mucosal regeneration and repair in intestinal goblet cells, where it is primarily co-secreted or co-expressed alongside MUC2, while it's expression is termed “intestinal”. By controlling tight junctions, TFF3 improves the function of the intestinal barrier by reducing the intestinal epithelium's paracellular permeability via regulation of tight junctions. This finding provide insight into the protective functions of TFF3 in epithelial cells and demonstrate its potential for treatment of inflammatory diseases in the GIT (10).

TFF3 suppresses the proliferation and differentiation of activated T cell subsets early during the process of CD4+ T cell differentiation. In recent times TFF3 has been recognized to affect liver metabolism and possible involvement in metabolic pathways through its action in improvement of glucose tolerance in diet-induced obesity model (53). Intestinal trefoil factor 3 (TFF3) protects and repairs the gastrointestinal mucosa and restores normal intestinal permeability, which is dependent on the integrity of the tight junction (TJ) barrier and the TJ associated proteins claudin-1, zona occludens-1 (ZO-1) and occludin (54). Mucus is essential to the gastrointestinal tract's integrity and for protecting epithelial cells from outside stimuli, infections, and mechanical damage (55). TFF3–FCGBP and MUC2 are the major components of intestinal mucus, and they both colocalize in the gut and play a key role in the innate immune defense of mucous epithelia (22). The combination of TFFs and mucin was effective in protecting the barrier function of epithelial cells. TFF3 promotes immunity and wound healing by de-repressing inhibitory LINGO2-EGFR complexes through interactions with LINGO2. TFF3 binds leucine rich repeat receptor and nogo-interacting protein 2 (LINGO2) to de-repress and enhance epidermal growth factor receptor protein (EGFR) signaling that drives wound healing and immunity against helminths (56).

## 2 Role of trefoil factors in protecting the gastrointestinal epithelium

Trefoil factors represent group of proteins with a crucial involvement in mucosal protection and repair processes. The complex interplay within the mucosal environment resulted in keen interest in understanding their functions in advancing treatments in gastrointestinal disease (57). This section unravels their biological significance and potential therapeutic implications in various animal species with a summary of actions in Table 1.

**Table 1:** Summary of expressions of intestinal trefoil factors in animals.

**Species**	**Treatment**	**Pre-condition**	**Action**	**References**
Rat	Wheat peptides (0.1, 0.2, 0.4 g/kgbw) and omeprazole (20 mg/kgbw)	–	Increasing trefoil factor 1 (TFF1) levels	(84)
Rat	Low dose administration of mature silkworm powder	Ethanol-induced gastric damage	Promoting genes related gastric mucosal protection and biosynthesis including mucin 5AC and trefoil factors. Induces gastric mucosal defense factors in ethanol-induced gastric injury rat model	(85)
Rodents	Astragalus polysaccharides and matrine treatment	Ulcerative colitis (UC)	Upregulating trefoil factor 3 expression	(49)
Rodents	Oral administration of TFF3	Hemotherapy- and radiotherapy-induced mucositis	Decreased the intestinal damage	(86)
Rodents	^1^Lingo2 mice	^1^200 *Trichuris muris* eggs/mouse by oral gavage	ITF(TFF3) restrains T_H_1 cell proliferation, promotes Type 2 immunity against infection	(87)
Rodents	Oxyresveratrol, an active ingredient of Artocarpus lakoocha administered to five treatment groups	Induction of gastric ulcer in mice	Elevated expression levels of cytoprotective TFF-2 levels, and attenuated expression levels of IL-6, TNF-α, NF-κB, and COX-2	(88)
Pigs	Control diet (supplemented 25 mg/kg quinocetone and 11.25 mg/kg aureomycin in the basal diet), three treatment diets supplemented with 200, 400, or 600 mg/kg bacteriophage in the basal diet	Healthy weaned piglets	ITF, and tumor growth factor-alpha (TGF-α) was enhanced	(89)
Pigs	Weaned piglets (28 D old)	Diarrhea occurrence (Enterotoxigenic *E. col*) Five levels of Wheat bran fiber or pea fiber.	Increased colonic peptide trefoil factors in piglets on the WBF and PF diets	(90)
Pigs	Weaned Piglets	Post-weaning (7 and 14 days) Dietary treatment [0, 200, or 400 μg/kg epidermal growth factor (EGF) supplementation].	Enhanced abundance of mucin 2 and intestinal trefoil factor 3 at 200 μg/kg EGF	(91)
Calves	Calves with coccidiosis received a single dose of toltrazuril (15 mg/kg) and supportive care	Control group (healthy calves) vs. Treatment group (calves with coccidiosis at 0th and 72nd hours infection)	Calves with coccidiosis had higher TFF-3 levels and ACTG2 after treatment (72nd hours) compared to the control group.	(91)
Cattle	Control group (negative controls)Positive control Orally infected with 10,000 Ostertagia ostertagi L3s (third-stage larva) and killed at 6, 9, and 24 days post infection (dpi)	Infection with nematode *Ostertagia ostertagi*	TFF1 and TFF3, co-expressed with mucins in the GI tract was upregulated in infected animals.	(46)
Cattle	Healthy cow, right displacement of the abomasum (RDA) and left displacement of the abomasum (LDA)	Displacement of abomasum	TFF-3 concentrations lower in RDA. Negative correlation between lactate and TFF-3	(92)
New-born Calves	Normal (NG) and test group (TG): *E. coli* O1(2.5 × 10^11^ CFU/mL, 100 mL)	Early pathogenic *Escherichia coli* infection	Lower ITF levels in the colons of TG calves than NG. Concentration of ITF in both the TG and NG decreased over experimental time	(23)
Sheep	^1^Group One (Chiswick Avermectin Resistant CAVRS) and Group two (ivermectin-sensitive McMaster)	*Haemonchus contortus* infection (two isolates: 500 McMaster L3 and 500 CAVR L3)	TFF3 was the second most highly upregulated gene after Intelectin 2 (IL-2) in the comparisons of day 22 vs. 3.	(93)
	Genetically Resistant group and Susceptible group	10,000 *H. contortus* L3s	TFF 2 was higher in resistant sheep	(94)
Sheep	Control group two (nfected with 30,000 T. *colubriformis* larvae L3) Group three: (infected with 10,000 H. *contortus* L3	Sheep infected with *Haemonchus contortus* and *Trichostrongylus columbriformis*	TFF3 was also found to be significantly upregulated in the small intestine (proximal jejunum), TFF2 was expressed in the abomasum.	(95)
Poultry	Arbor acre broilers	Supplementing paraformic acid (PFA)	Elevated intestinal mucosal factors (mucin 2, trefoil factor family, and zonula occludens-1) concentrations	(33)
	Arbor Acre broilers: basal diet [control (CON) group] and Tannic Acid (TA) group	Tannic acid extracted from *Galla chinensis* (300 mg/Kg TA in basal diet)	Intestinal mucosal ZO-1 and TFF expression was similar in both groups.	(96)
	Control (PBS-treated), AKK (orally administered 1 × 10^6^ CFU Akkermansia muciniphila for 10 days), SAL (orally administered 1 × 10^9^ CFU Salmonella pullorum-treated on the 5^th^ day) & AKK+SAL (orally administered 1 × 10^6^ CFU Akkermansia muciniphila for 10 days and orally administered 1 × 10^9^ CFU Salmonella pullorum-treated on the 5^th^ day).	*S. pullorum* (1 × 10^9^ CFU) and *Akkermansia muciniphila* (1 × 10^6^ CFU) gavaging of birds.	Increased number of goblet cells in the intestine and up-regulation of Mucin 2 and trefoil factor 2 (TFF2)	(97)
	Male Cobb 500	live *Eimeria* vaccination or salinomycin and combinations of in-feed inclusion of gelatin and vitamin E	TFF2, cytokines, and MUC2 was increased by *Eimeria* vaccination.	(98)
	SPF chickens (control group and infection group)	Avian Influenza Virus Subtype H9N2 (i noculated with 3 doses of A/Chicken/Henan/SH01/2015 at 10^6^ TCID_50_/0.1 mL through the respiratory tract	TFF2, MUC2, ZO-1, and caudin-3 expressions was downregulated by H9N2 AIV infection	(99)
	d-1 Cherry Valley ducks	0, 1, 2, 4, 8, and 16 mg/kg iodine (dietary ethylenediamine dihydroiodide) in diet	Similar expression level of TFF2 in treatment groups	(100)
	Arbor acre broilers CON (basal diet group), Group two (AB) Group three (BP)	Basal diet supplemented with 50 mg/kg aureomycin (AB group) and basal diet supplemented with 40 mg/kg Bopu powder (BP group)	Dietary Bopu powder supplementation increased concentration of trefoil factor family member and mRNA expressions of superoxide dismutase	(101)
	Fertile Cobb 500 eggs	Eggs Injected in the amnion with 100 μL of either sterile water (sham), 1 × 10^5^, 1 × 10^6^, or 1 × 10^7^ (P1, P2, and P3) probiotic product (Primalac W/S containing *Lactobacillus acidophilus, Lactobacillus casei, Enterococcus faecium*, and *Bifidobacterium bifidum*)	In ovo probiotic supplementation was associated with downregulated expression of Toll-like receptors-2 and−4, inducible nitric oxide synthase, trefoil factor 2, mucin-2, interferon-γ, and interleukins-4 and−13 in both the ileum and cecal tonsils	(102)

### 2.1 Trefoil factors on the gut health of pigs/piglets

Intestinal epithelial cells form the basic unit of the gastrointestinal tract, and therefore play a unique role of maintaining the integrity of the mucosal barrier, nutrient absorption, and disease prevention (57). In the GIT of pigs, TFFs functions in the establishment of mucosal protection and repair. Although TFF1 is mainly expressed in the pyloric gland and the neck cells of the stomach, all TFFs are abundantly produced in the goblet cells—a mucin-producing epithelial cell in the small and large intestines (2). In general, the intestinal epithelial cells in the gut of pigs contribute to maintaining the defense and integrity of the mucosal layer (58). In general, TFF peptides maintain intestinal epithelial integrity in various organisms through restitution, wound healing, apoptosis, cell motility, and as well as establishing the protective effects of the intestinal barrier (59, 60). During mucosal repair, both pro and anti-inflammatory cytokines regulate the actions of TFFs (61). For instance, during intestinal development in pigs, transforming growth factor-alpha (TGF-α, a cytokine) modulates TFF2 and TFF3 to exhibit different regulation patterns (36, 62). Accordingly, TFF2 expression was significantly upregulated after weaning phase, and this in-turn enhanced mucosal integrity. During the pre-weaning phase in piglets, TFFs such as TFF3 may facilitate intestinal repair of injury induced by inflammation, thereby enhancing intestinal development and growth performance (50). The ability of TFFs to rebuild (or repair) the intestinal epithelium has been attributed to their anti-apoptotic mechanisms that drive the migration of epithelial cells over the damaged section of the intestinal mucosa, where the apoptosis is induced by anchorage-dependent cells detaching from the surrounding extracellular matrix (52) in pigs. In addition, TFF3 has also been reported to suppress factors that downregulate tight junction proteins, thereby decreasing mucosal permeability, promoting the integrity of tight junctions, and consequently enhancing intestinal barrier function (63).

### 2.2 Trefoil factors on the gut health in chicken

Although the molecular mechanisms underlying the functions of TFFs has been well-explored in mammalian species like rodents, humans and other amphibians, information is scanty regarding avian species (64). It has been established that the protective effect of TFFs result from their interactions with mucins to generate signaling mechanisms that result in their overexpression during mucosal damage, consequently culminating in intestinal restitution of the mucosa and enhanced barrier function (65). A similar mechanism of action has been reported in the chicken, and this was characterized by migration of the epithelial cells to the epithelial layers of the intestinal villi during injury (50). TFF2 bearing one single trefoil domain promote cell migration (45), since a damaged intestinal mucosa encourages the migration of pathogens into the body thereby causing systemic infection, trefoil factors mediate as a host defense mechanism by keeping the gastrointestinal mucosal layer impermissible to pathogenic attack (66). Accordingly, it can be proposed that the activities of TFFs inhibit pathogenic infection in concert with the actions of other antimicrobial peptides. This notion is corroborated by the finding that chickens infected with avian influenza (H9N2) showed significant decrease in the expression of TFFs and other antimicrobial peptides, and consequently, this resulted in infection with E. coli bacteria (67).

### 2.3 Trefoil factors on the gut health in rodents

The fact that most of the studies investigating the role of TFFs in gastrointestinal health were done in rodents warrant the exploration of literature in this regard in mice. Several studies have demonstrated the protective and healing effects of TFFs following mucosal damage in rodents (68). Conversely, anomalies develop in mucosal layer when there is deficiency of TFFs in mice. Studies have shown that TFF-2 deficient mice showed high susceptibility to gastric injury, and TFF-3 deficient mice showed a reduced resistance to colonic injury (69, 70). Mode of action of TFFs involve the formation of mucous defense and barriers, modulation of the mucosal immune response as well as enhancing a rapid mucosal repair via cell migration; a process known as restitution (71). The restitution process is a pivotal mechanism following a mucosal damage as it helps in mucosal repair by migrating neighboring cells to the injury sites (71). This is evident in previous research reporting that TFF3 restitutes epithelial cells by stimulating the migration of cells surrounding the injured area to the damaged site (73). In addition, TFFs also exhibit antiapoptotic effects by rapidly preventing cell death through maintaining cell migration and cell survival (71). A research study conducted by Sturm and Dignass (72) showed that TFF3 promotes migration of epithelial cells by influencing localization and expression of catenin in epithelial cells, and by stimulating phosphorylation of catenin. The expression of TFF2 and TFF3 have also been observed to simultaneously induce the migration of monocytes in the bone marrow, lymph nodes, thymus and other lymphoid tissues (71). It has been established that the inflammatory responses of immune systems of mice is dysfunctional when TFF2 is mostly deficient (15), thus confirming that TFF2 is closely associated with immune response in addition to its involvement in gastric repair of epithelial cells. The phosphatidylinositol 3′-kinase (PI3K/Akt) signaling pathway which is activated by ITF, serves as a regulatory intracellular avenue that restores the epithelial mucosal integrity following an injury (74).

Many studies have confirmed that the PI3K/Akt signaling pathway actively participates in key physiological and pathological processes such as regulating cell proliferation, migration and apoptosis, as well as exhibiting inflammatory responses in the epithelial mucosa (75, 76). For instance, it was observed that higher concentration of ITF increased the proliferation and migration of gut esterase-1 (GES-1) cells (74), thereby corroborating the findings of previous studies (77, 78). In the large intestine, the absence of TFF3 expression was found to increase sensitivity to colonic injury by stabilizing the mucosal layer and inducing repair at injury sites, and rapidly upregulating and enhancing the restorative process (6). This protective effect may be attributed to the collaborative action of TFF3 and Muc2 in the intestinal mucosa as the first line of defense against epithelial injury (79). It has also been reported that recombinant human TFF3 (rhTFF3) can stimulate the expression of tight junction proteins which are directly connected with intestinal barrier functions, thereby reducing intestinal mucosal permeability (74). This was evident in an rhTFF3 expression study on intestinal wall injury in the rat (80). In this study, Wang et al. (80) observed a significant reduction in damage to ileal mucosa, and a reduced intestinal permeability.

### 2.4 Trefoil factors on the gut health in calves

The intestinal mucosa is crucial for regulating the interaction between bacteria and host cells, as well as affecting nutrient absorption. Calves are mostly susceptible to numerous mechanical obstructions as a result of abnormalities in the bowel lumen, intestinal wall, or outside the intestinal tract (81). Calves, like other young animals, experience gastrointestinal injuries or insults due to factors such as weaning stress, infections, or dietary imbalances. Trefoil factors, particularly TFF2 and TFF3 has been established to play a significant role in maintaining and repairing the intestinal mucosa in calves (23). Studies have shown that trefoil factors like TFF3 can help prevent intestinal epithelial damage and promote repair of the intestinal mucosa. In calves, this protection is particularly crucial during the early stages of life when gastrointestinal system is still developing and vulnerable to various stressors, including dietary changes and pathogens. The immunological barrier function mediated by intestinal microbes is destroyed by changes in the makeup of the intestinal flora in calves, making the intestinal tract more vulnerable to harmful germs and putting the animals' health at risk (82). The abomasal infection by nematodes in calves such as *Ostertagia ostertagi* larvae causes substantial tissue injury to the abomasal mucosa, and these injuries include acute epithelial cytolysis, hyperplasia of gastric glands, and reduction in acid-producing cells, all culminating in increased gastric pH concentration and inhibition of protein metabolism (83). In another study, the mucin glucosaminyl (N-acetyl) transferase 3 which catalyzes one of the key rate-limiting steps in mucin biosynthesis was up-regulated in the bovine abomasum during nematode infections (46). Together, these results suggest TFF-induced enhanced tissue repair and mucin secretion may contribute directly to mucosal protective immunity. It can be suggested that TFFs enhance mucosal defense by inducing tissue repair mechanisms in the GIT through stimulating cell movement in a manner that reestablishes a healthy mucosa and inhibit apoptosis (84). Intestinal biomarkers have been found to be elevated in inflammatory bowel damage and acute intestinal ischemia (blood flow to the intestine), and intestinal mucosal injury has been linked to elevated expression levels of IFABP, LFABP, IAP, and TFF3 (81).

## 3 Feed additives: TFFs activation

TFFs play critical roles in maintaining the integrity and repair of mucosal barriers, particularly in the gastrointestinal tract (1). Certain feed additives and nutrients have been studied for their ability to stimulate or enhance the expression of TFFs, potentially improving gut health and promoting healing. Some feed additives that can stimulate or activate trefoil factors are listed in Table 2.

**Table 2:** Feed additives and their impact on trefoil factor (TFF) activation in animal models.

**Additive**	**Treatment**	**Action**	**References**
Lactic acid bacteria (LAB)	LAB-fermented formula milk supplementation given to weaned piglets	Increased the average daily gain, UP-regulated TFF2, selective enrichment of lactate-producing and short-chain fatty acid (SCFA)-producing bacteria in the ileum	(103)
*Lactobacillus rhamnosus*	Newborn piglets were orally administered with Wild-type (WT) and mutant (ΔluxS) *Lactobacillus rhamnosus*, Control (CON)	ITF levels were increased in the WT and ΔluxS group than in the CON group	(104)
Epidermal growth factor	Dietary treatment (0, 200, or 400 μg/kg EGF supplementation)	Dietary supplementation with 200 μg/kg enhanced abundances of mucin 2 and intestinal TFF3 compared with the control	(91)
Dietary Fiber	Control diet without fiber source (CT), and diets: maize was replaced by 10 % maize fiber (MF), 10 % soyabean fiber (SF), 10 % wheat bran fiber (WBF) or 10 % pea fiber (PF).	WB and PF resulted in increased expression of transforming growth factor-alpha (TGF-α), trefoil factors.	(90)
Dietary crude protein	Weanling pigs fed Three (3) diets containing 22, 19, or 16% CP	A reduction in occludin, trefoil factor-2, trefoil factor-3, and mucin 2 was observed at 16% CP	(105)
Zearalenone (ZEA)	Diet supplemented with ZEA at 0, 0.15, 1.5, and 3.0 mg/kg fed to weaned piglets	ZEA at 0.15 upregulated TFF3	(60)
Probiotic mixture	Probiotic mixture VSL containing four species of *Lactobacilli*: three species of *Bifidobacteria* and *Streptococcus salivarius* subsp. *thermophilus* sp.	Increased production of mucus, TFF1 and TFF2	(106)
Taurine	Lipopolysaccharide (LPS) and dietary taurine	Taurine increased the expression of TFF-3 mRNA. LPS-challenged animals lowered expressions of trefoil factor-3, transforming growth factor β-1 expression and number of goblet cells	(107)
Palygorskite	Basal diet supplemented with 0, 5, and 10 g/kg palygorskite for 21 days in chicks	Supplementation of 5 g/kg palygorskite increased jejunal trefoil factor 2 (TFF2) mRNA abundance	(108)
*Lactiplantibacillus plantarum*	Negative control (NC): basal diet only; OTC: basal diet + 0.01% oxytetracycline; RG11: basal diet + 0.1% Postbiotic RG11; RI11: basal diet + 0.1% postbiotics RI11 and RS5: basal diet + 0.1% postbiotic RS5	The concentrations of acidic mucin, sulfated mucin, and intestinal trefoil factor were higher in the birds fed with RI11 and RS5.	(109)
Paraformic acid	Basal diet (CON) or a basal diet supplemented with 1,000 mg/kg PFA	PFA supplementation elevated intestinal mucosal factors (mucin 2, trefoil factor family, and zonula occludens-1)	(33)
Yeast nucleotides	Control (CON = basal diet), groups 2–4 were fed the basal diet supplemented with 0.1, 0.3, and 0.5% yeast nucleotides	0.1 and 0.3% yeast nucleotides exhibited higher expression of Mucin 2 (MUC2) and trefoil factor 2 (TFF2) gene	(110)
Probiotics (Primalac W/S)	Probiotics (Primalac: 1 × 10^5^, 1 × 10^6^, or 1 × 10^7^ (P1, P2, and P3 probiotic bacteria.) and Sham *in ovo* injection of Cobb500 eggs	Probiotic supplementation downregulated expression of Toll-like receptors-2 and−4, inducible nitric oxide synthase, TFF2, mucin-2, interferon-γ, and interleukins-4 and−13 in both the ileum and cecal tonsils.	(102)
*Macleaya Cordata* (Bopu powder)	Basal diet (CON group), basal diet supplemented with 50 mg/kg aureomycin (AB group), or a basal diet supplemented with 40 mg/kg Bopu powder (BP group)	Dietary Bopu powder supplementation significantly increased the concentration of trefoil factor family member	(101)
Algae	Seaweed extract (SWE; 10.0 g/d) and fish oil (FO; 100 g/d) inclusion	SWE supplementation increase colonic TFF3 mRNA expression	(111)
*Bifidobacterium bifidum*	Milk formula containing 5 × 10^6^ CFU per day of *Bifidobacterium bifidum* OLB6378 and dam-fed littermates fed by surrogate mothers as a baseline control	*B. bifidum* treatment of neonatal necrotizing enterocolitis (NEC) markedly reduced number of Tff3-positive cells to values seen in normal healthy controls.	(112)
Cinnamaldehyde	Treatment with cinnamaldehyde (100 or 200 mg/kg bodyweight/day)	Cinnamaldehyde upregulated the expression of TFF3	(113)

## 4 Conclusion

The GIT tracts of animals is often exposed to injuries, proliferation of pathogenic bacterial, stress caused by chemicals, physical stress and also adverse effects related to side effects of medical drugs used as growth promoters, consequently necessitating the need for tissue repairs. Various studies have indicated that TFFs enhanced protection and restitution of the mucosal surfaces of various vital organs, including the GIT. The beneficial effects of TFFs are achieved through molecular interactions with mucins to improve cellular migration. The findings from this literature exploration confirms that TFF peptides play significant beneficial roles in intestinal epithelial healing and amalgamation of the mucus layer. Such protection of the intestinal epithelial barrier is prone to culminate in improved animal health, growth performance, and production. Accordingly, future research should be conducted to identify biogenic feed additives that have potential to induce optimum synthesis of TFFs in the GIT of food animals.

## References

[1] Gomez-Osorio L Yepes-Medina V Ballou A Parini M Angel R. Short and medium chain fatty acids and their derivatives as a natural strategy in the control of necrotic enteritis and microbial homeostasis in broiler chickens. Front Vet Sci. (2021) 8:773372. 10.3389/fvets.2021.773372 34970616 PMC8712453

[2] Xiao P Ling H Lan G Liu J Hu H Yang R. Trefoil factors: gastrointestinal-specific proteins associated with gastric cancer. Clin Chim Acta. (2015) 450:127–34. 10.1016/j.cca.2015.08.004 26265233

[3] Lin J Sun Z Zhang W Liu H Shao D Ren Y. Protective effects of intestinal trefoil factor (ITF) on gastric mucosal epithelium through activation of extracellular signal-regulated kinase 1/2 (ERK1/2). Mol Cell Biochem. (2015) 404:263–70. 10.1007/s11010-015-2386-2 25776570

[4] Gomez-Osorio LM Jiang Z Zhang Q Yan H Villegas AM Applegate T. Secretory defense response in the bird's gastro-intestinal tract and nutritional strategies to modulate it. In: Patra AK, editor. Advances in Poultry Nutrition Research. London: IntechOpen (2021).

[5] Baus-Loncar M Giraud AS. Multiple regulatory pathways for trefoil factor (TFF) genes. Cell Mol Life Sci. (2005) 62:2921. 10.1007/s00018-005-5480-x 16374580 PMC11139188

[6] Marchbank T Playford RJ. Trefoil factor family peptides enhance cell migration by increasing cellular osmotic permeability and aquaporin 3 levels. FASEB J. (2018) 32:1017–24. 10.1096/fj.201700799R 29046361

[7] Masiakowski P Breathnach R Bloch J Gannon F Krust A Chambon P. Cloning of cDNA sequences of hormone-regulated genes from the MCF-7 human breast cancer cell line. Nucleic Acids Res. (1982) 10:7895–903. 10.1093/nar/10.24.7895 6897676 PMC327057

[8] Jorgensen KH Thim L Jacobsen HE. Pancreatic spasmolytic polypeptide (PSP): I. Preparation and initial chemical characterization of a new polypeptide from porcine pancreas. Regul Pept. (1982) 3:207–19. 10.1016/0167-0115(82)90126-4 6896240

[9] Suemori S Lynch-Devaney K Podolsky DK. Identification and characterization of rat intestinal trefoil factor: tissue-and cell-specific member of the trefoil protein family. Proc Nat Acad Sci USA. (1991) 88:11017–21. 10.1073/pnas.88.24.11017 1763017 PMC53064

[10] Aihara E Engevik KA Montrose MH. Trefoil factor peptides and gastrointestinal function. Annu Rev Physiol. (2017) 79:357–80. 10.1146/annurev-physiol-021115-105447 27992733 PMC5474939

[11] Thim L Madsen F Poulsen SS. Effect of trefoil factors on the viscoelastic properties of mucus gels. Eur J Clin Invest. (2002) 32:519–27. 10.1046/j.1365-2362.2002.01014.x 12153553

[12] Playford RJ Marchbank T Goodlad RA Chinery RA Poulsom R Hanby AM. Transgenic mice that overexpress the human trefoil peptide pS2 have an increased resistance to intestinal damage. Proc Nat Acad Sci USA. (1996) 93:2137–42. 10.1073/pnas.93.5.2137 8700898 PMC39923

[13] Farrell JJ Taupin D Koh TJ Chen D Zhao C-M Podolsky DK. TFF2/SP-deficient mice show decreased gastric proliferation, increased acid secretion, and increased susceptibility to NSAID injury. J Clin Invest. (2002) 109:193–204. 10.1172/JCI12529 11805131 PMC150833

[14] Hoffmann W Jagla W. Cell type specific expression of secretory TFF peptides: colocalization with mucins and synthesis in the brain. Int Rev Cytol. (2002) 213:147–81. 10.1016/S0074-7696(02)13014-2 11837892

[15] Kurt-Jones EA Cao L Sandor F Rogers AB Whary MT Nambiar PR. Trefoil family factor 2 is expressed in murine gastric and immune cells and controls both gastrointestinal inflammation and systemic immune responses. Infect Immun. (2007) 75:471–80. 10.1128/IAI.02039-05 17101660 PMC1828407

[16] Regalo G Wright NA Machado JC. Trefoil factors: from ulceration to neoplasia. Cell Mol Life Sci. (2005) 62:2910–5. 10.1007/s00018-005-5478-4 16374578 PMC11138378

[17] Hoffmann W. TFF (trefoil factor family) peptides and their potential roles for differentiation processes during airway remodeling. Curr Med Chem. (2007) 14:2716–9. 10.2174/092986707782023226 17979720

[18] Kinoshita K Taupin DR Itoh H Podolsky DK. Distinct pathways of cell migration and antiapoptotic response to epithelial injury: structure-function analysis of human intestinal trefoil factor. Mol Cell Biol. (2000) 20:4680–90. 10.1128/MCB.20.13.4680-4690.2000 10848594 PMC85884

[19] Efstathiou JA Noda M Rowan A Dixon C Chinery R Jawhari A. Intestinal trefoil factor controls the expression of the adenomatous polyposis coli–catenin and the E-cadherin–catenin complexes in human colon carcinoma cells. Proc Nat Acad Sci USA. (1998) 95:3122–7. 10.1073/pnas.95.6.3122 9501226 PMC19705

[20] Hensel KO Boland V Postberg J Zilbauer M Heuschkel R Vogel S. Differential expression of mucosal trefoil factors and mucins in pediatric inflammatory bowel diseases. Sci Rep. (2014) 4:7343. 10.1038/srep07343 25475414 PMC4256710

[21] Chen X Hu Y Xie Y Wang Y. High salt diet can down-regulate TFF2 expression level in gastric mucosa of MGs after *H. pylori* infection. Microbial Pathog. (2018) 118:316–21. 10.1016/j.micpath.2018.03.047 29601867

[22] Schumacher MA Danopoulos S Al Alam D Frey MR. Growth factors in the intestinal tract. In: Said HM, editor. Physiology of the Gastrointestinal Tract. Elsevier (2018). p. 71–101.

[23] He L Wang C Simujide H Aricha H Zhang J Liu B. Effect of early pathogenic escherichia coli infection on the intestinal barrier and immune function in newborn calves. Front Cell Infect Microbiol. (2022) 12:818276. 10.3389/fcimb.2022.818276 35265533 PMC8900010

[24] Zhang B Yu H Sheng Z Luo H Yu J. The therapeutic effect of recombinant human trefoil factor 3 on hypoxia-induced necrotizing enterocolitis in immature rat. Regul Pept. (2003) 116:53–60. 10.1016/S0167-0115(03)00177-0 14599715

[25] Jørgensen KD Diamant B Jørgensen KH Thim L. Pancreatic spasmolytic polypeptide (PSP): III. pharmacology of a new porcine pancreatic polypeptide with spasmolytic and gastric acid secretion inhibitory effects. Regul Pept. (1982) 3:231–43. 10.1016/0167-0115(82)90128-8 6919177

[26] Braga Emidio N Brierley SM Schroeder CI Muttenthaler M. Structure, function, therapeutic potential of the trefoil factor family in the gastrointestinal tract. ACS Pharmacol Transl Sci. (2020) 3:583–97. 10.1021/acsptsci.0c00023 32832864 PMC7432662

[27] Wright NA Hoffmann W Otto WR Rio M Thim L. Rolling in the clover: trefoil factor family (TFF)-domain peptides, cell migration and cancer. FEBS Lett. (1997) 408:121–3. 10.1016/S0014-5793(97)00424-9 9187350

[28] Kuemmerle JF Barnard JA McHugh KM. Growth factors in the gastrointestinal tract. In: Barrett KE Ghishan FK Ju, editors. Physiology of the Gastrointestinal Tract. Elsevier (2012). p. 199–277.

[29] Thim L. A new family of growth factor-like peptides ‘Trefoil' disulphide loop structures as a common feature in breast cancer associated peptide (pS2), pancreatic spasmolytic polypeptide (PSP), and frog skin peptides (spasmolysins). FEBS Lett. (1989) 250:85–90. 10.1016/0014-5793(89)80690-8 2737304

[30] Nunez A Jakowlev S Briand J Gaire M Krust A Rio M. Characterization of the estrogen-induced pS2 protein secreted by the human breast cancer cell line MCF-7. Endocrinology. (1987) 121:1759–65. 10.1210/endo-121-5-1759 3665845

[31] Thim L Thomsen J Christensen M Jørgensen KH. The amino acid sequence of pancreatic spasmolytic polypeptide. Biochim Biophys Acta. (1985) 827:410–8. 10.1016/0167-4838(85)90226-2 2857575

[32] Hoffmann W. Trefoil factor family (tff) peptides and their links to inflammation: a re-evaluation and new medical perspectives. Int J Mol Sci. (2021) 22:4909. 10.3390/ijms22094909 34066339 PMC8125380

[33] Li J Liu Y Niu J Jing C Jiao N Huang L. Supplementation with paraformic acid in the diet improved intestinal development through modulating intestinal inflammation and microbiota in broiler chickens. Front Microbiol. (2022) 13:975056. 10.3389/fmicb.2022.975056 36204610 PMC9531753

[34] Familari M Cook GA Taupin DR Marryatt G Yeomans ND Giraud AS. Trefoil peptides are early markers of gastrointestinal maturation in the rat. Int J Dev Biol. (1998) 42:783–9. 9727834

[35] Kuemmerle JF Barnard JA McHugh KM. Chapter 8 - growth factors in the gastrointestinal tract. In:Johnson LR Ghishan FK Kaunitz JD Merchant JL Said HM Wood JD, editors. Physiology of the Gastrointestinal Tract, 5th ed. Boston, MA: Academic Press (2012) 199–277.

[36] Baus-Loncar M Lubka M Pusch CM Otto WR Poulsom R Blin N. Cytokine regulation of the trefoil factor family binding protein GKN2 (GDDR/TFIZ1/blottin) in human gastrointestinal epithelial cells. Cell Physiol Biochem. (2006) 20:193–204. 10.1159/000104166 17595528

[37] Nikolaidis NM Zimmermann N King NE Mishra A Pope SM Finkelman FD. Trefoil factor-2 is an allergen-induced gene regulated by Th2 cytokines and STAT6 in the lung. Am J Respir Cell Mol Biol. (2003) 29:458–64. 10.1165/rcmb.2002-0309OC 12702542

[38] Podolsky DK Gerken G Eyking A Cario E. Colitis-associated variant of TLR2 causes impaired mucosal repair because of TFF3 deficiency. Gastroenterology. (2009) 137:209–20. 10.1053/j.gastro.2009.03.007 19303021 PMC2812790

[39] Yuan F Zhou Z Wu S Jiao F Chen L Fang L. Intestinal activating transcription factor 4 regulates stress-related behavioral alterations via paraventricular thalamus in male mice. Proc Nat Acad Sci USA. (2023) 120:e2215590120. 10.1073/pnas.2215590120 37126693 PMC10175747

[40] Soutto M Chen Z Bhat AA Wang L Zhu S Gomaa A. Activation of STAT3 signaling is mediated by TFF1 silencing in gastric neoplasia. Nat Commun. (2019) 10:3039. 10.1038/s41467-019-11011-4 31292446 PMC6620282

[41] Znalesniak EB Salm F Hoffmann W. Molecular alterations in the stomach of Tff1-deficient mice: early steps in antral carcinogenesis. Int J Mol Sci. (2020) 21:644. 10.3390/ijms21020644 31963721 PMC7014203

[42] Otto WR Thim L. Trefoil factors: trefoil factor family-interacting proteins. Cell Mol Life Sci. (2005) 62:2939–46. 10.1007/s00018-005-5482-8 16374582 PMC11139177

[43] Heuer J Heuer F Stürmer R Harder S Schlüter H Braga Emidio N. The tumor suppressor TFF1 occurs in different forms and interacts with multiple partners in the human gastric mucus barrier: Indications for diverse protective functions. Int J Mol Sci. (2020) 21:2508. 10.3390/ijms21072508 32260357 PMC7177788

[44] Kim O Yoon JH Choi WS Ashktorab H Smoot DT Nam SW. Heterodimeric interaction between GKN2 and TFF1 entails synergistic antiproliferative and pro-apoptotic effects on gastric cancer cells. Gastric Cancer. (2017) 20:772–83. 10.1007/s10120-017-0692-y 28150071 PMC5718056

[45] Jiang Z Lossie AC Applegate TJ. Evolution of trefoil factor(s): genetic and spatio-temporal expression of trefoil factor 2 in the chicken (gallus gallus domesticus). PLoS ONE. (2011) 6:e22691. 10.1371/journal.pone.0022691 21829480 PMC3146476

[46] Rinaldi M Dreesen L Hoorens PR Li RW Claerebout E Goddeeris B. Infection with the gastrointestinal nematode ostertagia ostertagi in cattle affects mucus biosynthesis in the abomasum. Vet Res. (2011) 42:1–11. 10.1186/1297-9716-42-61 21569362 PMC3102617

[47] Heuer F Stürmer R Heuer J Kalinski T Lemke A Meyer F. Different forms of TFF2, a lectin of the human gastric mucus barrier: *in vitro* binding studies. Int J Mol Sci. (2019) 20:5871. 10.3390/ijms20235871 31771101 PMC6928932

[48] Šešelja K Bazina I Vrecl M Farger J Schicht M Paulsen F. Tff3 deficiency differentially affects the morphology of male and female intestines in a long-term high-fat-diet-fed mouse model. Int J Mol Sci. (2023) 24:16342. 10.3390/ijms242216342 38003531 PMC10671422

[49] Yan X Lu Q Zeng L Li X Liu Y Du X. Synergistic protection of astragalus polysaccharides and matrine against ulcerative colitis and associated lung injury in rats. World J Gastroenterol. (2020) 26:55. 10.3748/wjg.v26.i1.55 31933514 PMC6952295

[50] Taupin D Podolsky DK. Trefoil factors: initiators of mucosal healing. Nat Rev Mol Cell Biol. (2003) 4:721–32. 10.1038/nrm1203 14506475

[51] Hauser F Poulsom R Chinery R Rogers LA Hanby AM Wright NA. hP1. B, a human P-domain peptide homologous with rat intestinal trefoil factor, is expressed also in the ulcer-associated cell lineage and the uterus. Proc Natl Acad Sci. (1993) 90:6961–5. 10.1073/pnas.90.15.6961 8346203 PMC47055

[52] Li HP Xu CM Wen BY Li AQ Zha GM Jin XY. Extracellular production of recombinant sus scrofa trefoil factor 3 by *Brevibacillus choshinensis*. Exp Ther Med. (2020) 19:2149–54. 10.3892/etm.2020.8477 32104278 PMC7027283

[53] Xue Y Shen L Cui Y Zhang H Chen Q Cui A. Tff3, as a novel peptide, regulates hepatic glucose metabolism. PLoS ONE. (2013) 8:e75240. 10.1371/journal.pone.0075240 24086476 PMC3781022

[54] Lin N Xu L Sun M. The protective effect of trefoil factor 3 on the intestinal tight junction barrier is mediated by toll-like receptor 2 via a PI3K/akt dependent mechanism. Biochem Biophys Res Commun. (2013) 440:143–9. 10.1016/j.bbrc.2013.09.049 24051092

[55] Huang Y Wang M Yang Z Ren Y Zhang W Sun Z. Pretreatment with intestinal trefoil factor alleviates stress-induced gastric mucosal damage via akt signaling. World J Gastroenterol. (2020) 26:7619. 10.3748/wjg.v26.i48.7619 33505140 PMC7789054

[56] Belle NM Ji Y Herbine K Wei Y Park J Zullo K. TFF3 interacts with LINGO2 to regulate EGFR activation for protection against colitis and gastrointestinal helminths. Nat Commun. (2019) 10:4408. 10.1038/s41467-019-12315-1 31562318 PMC6764942

[57] Stürmer R Müller S Hanisch FG Hoffmann W. Porcine gastric TFF2 is a mucus constituent and differs from pancreatic TFF2. Cell Physiol Biochem. (2014) 33:895–904. 10.1159/000358662 24713603

[58] Wang S Zhang C Wang X Yang J Wu K Zhang J. Deoxynivalenol inhibits porcine intestinal trefoil factors expression in weanling piglets and IPEC-J2 cells. Toxins. (2019) 11:670. 10.3390/toxins11110670 31731782 PMC6891430

[59] Saliu EM Martínez-Vallespín B Aschenbach JR Brockmann GA Fulde M Hartmann S. Dietary fiber and its role in performance, welfare, and health of pigs. Anim Health Res Rev. (2022) 23:165–93. 10.1017/S1466252322000081 36688278

[60] Zhang P Jing C Liang M Jiang S Huang L Jiao N. Zearalenone exposure triggered cecal physical barrier injury through the TGF-β1/Smads signaling pathway in weaned piglets. Toxins. (2021) 13:902. 10.3390/toxins13120902 34941739 PMC8708673

[61] Lee SI Kim IH. Nucleotide-mediated SPDEF modulates TFF3-mediated wound healing and intestinal barrier function during the weaning process. Sci Rep. (2018) 8:4827. 10.1038/s41598-018-23218-4 29555969 PMC5859294

[62] Scholven J Taras D Sharbati S Schön J Gabler C Huber O. Intestinal expression of TFF and related genes during postnatal development in a piglet probiotic trial. Cell Physiol Biochem. (2009) 23:143–56. 10.1159/000204103 19255509

[63] Xu LF Teng X Guo J Sun M. Protective effect of intestinal trefoil factor on injury of intestinal epithelial tight junction induced by platelet activating factor. Inflammation. (2012) 35:308–15. 10.1007/s10753-011-9320-x 21452036

[64] Hnia K Notarnicola C Santa Barbara Pde Hugon G Rivier F. Biochemical properties of gastrokine-1 purified from chicken gizzard smooth muscle. PLoS ONE. (2008) 3:e3854. 10.1371/journal.pone.0003854 19057650 PMC2588339

[65] Suzuki S Hayama M Nakamura M Yamauchi K Maruta F Miyagawa S. Trefoil factor 2 in gland mucous cell mucin in the mucous gel covering normal or damaged gastric mucosa using the mongolian gerbil model. Scand J Gastroenterol. (2006) 41:1390–7. 10.1080/00365520600792077 17101569

[66] Chappell L Kaiser P Barrow P Jones MA Johnston C Wigley P. The immunobiology of avian systemic salmonellosis. Vet Immunol Immunopathol. (2009) 128:53–9. 10.1016/j.vetimm.2008.10.295 19070366

[67] Li H Liu X Chen F Zuo K Wu C Yan Y. Avian influenza virus subtype H9N2 affects intestinal microbiota, barrier structure injury, and inflammatory intestinal disease in the chicken ileum. Viruses. (2018) 10:270. 10.3390/v10050270 29783653 PMC5977263

[68] Hoffmann W. TFF (trefoil factor family) peptides. In: Kastin AJ, editors. Handbook of Biologically Active Peptides. San Diego, CA: Elsevier (2006). p. 1147–54.

[69] Farrell JJ Taupin D Koh TJ Chen D Zhao CM Podolsky DK. TFF2/SP-deficient mice show decreased gastric proliferation, increased acid secretion, and increased susceptibility to NSAID injury. J Clin Invest. (2002) 109:193–204. 10.1172/JCI0212529 11805131 PMC150833

[70] Mashimo H Wu DC Podolsky DK Fishman MC. Impaired defense of intestinal mucosa in mice lacking intestinal trefoil factor. Science. (1996) 274:262–5. 10.1126/science.274.5285.262 8824194

[71] Hoffmann W. TFF-triggered signals promoting restitution of mucous epithelia. Cell Mol Life Sci. (2005) 62:2932–8. 10.1007/s00018-005-5481-9 16374581 PMC11139160

[72] Sturm A Dignass AU. Epithelial restitution and wound healing in inflammatory bowel disease. World J Gastroenterol. (2008) 14:348–53. 10.3748/wjg.14.348 18200658 PMC2679124

[73] Perrais M Chen X Perez-Moreno M Gumbiner BM. E-cadherin homophilic ligation inhibits cell growth and epidermal growth factor receptor signaling independently of other cell interactions. Mol Biol Cell. (2007) 18:2013–25. 10.1091/mbc.e06-04-0348 17392517 PMC1877107

[74] Sun Z Liu H Yang Z Shao D Zhang W Ren Y. Intestinal trefoil factor activates the PI3K/Akt signaling pathway to protect gastric mucosal epithelium from damage. Int J Oncol. (2014) 45:1123–32. 10.3892/ijo.2014.2527 24990304

[75] Li Q Zhu GD. Targeting serine/threonine protein kinase B/Akt and cell-cycle checkpoint kinases for treating cancer. Curr Top Med Chem. (2002) 2:939–71. 10.2174/1568026023393318 12171565

[76] Bao S Wang Y Sweeney P Chaudhuri A Doseff AI Marsh CB. Keratinocyte growth factor induces Akt kinase activity and inhibits Fas-mediated apoptosis in A549 lung epithelial cells. Am J Physiol Lung Cell Mol Physiol. (2005) 288:L36–42. 10.1152/ajplung.00309.2003 15347568

[77] Zheng Q Gao J Li H Guo W Mao Q Gao E. Trefoil factor 3 peptide regulates migration via a Twist-dependent pathway in gastric cell. Biochem Biophys Res Commun. (2013) 438:6–12. 10.1016/j.bbrc.2013.06.115 23845905

[78] Qu Y Yang Y Ma D Xiao W. Increased trefoil factor 3 levels in the serum of patients with three major histological subtypes of lung cancer. Oncol Rep. (2012) 27:1277–83. 10.3892/or.2012.1627 22246423 PMC3583529

[79] Michielan A D'Incà R. Intestinal permeability in inflammatory bowel disease: pathogenesis, clinical evaluation, and therapy of leaky gut. Mediat Inflammat. (2015) 2015:628157. 10.1155/2015/628157 26582965 PMC4637104

[80] Wang Y Liang K Kong W. Intestinal trefoil factor 3 alleviates the intestinal barrier function through reducing the expression of TLR4 in rats with nonalcoholic steatohepatitis. Arch Med Res. (2019) 50:2–9. 10.1016/j.arcmed.2019.03.004 31101239

[81] Yildiz R Ok M Ider M Aydogdu U Naseri A Parlak K. Evaluation of intestinal damage biomarkers in calves with atresia coli. J Vet Res. (2018) 62:379–84. 10.2478/jvetres-2018-0054 30584620 PMC6295999

[82] Li RW Hou Y Li C Gasbarre LC. Localized complement activation in the development of protective immunity against *Ostertagia ostertagi* infections in cattle. Vet Parasitol. (2010) 174:247–56. 10.1016/j.vetpar.2010.08.037 20884121

[83] Taupin DR Kinoshita K Podolsky DK. Intestinal trefoil factor confers colonic epithelial resistance to apoptosis. Proc Natl Acad Sci USA. (2000) 97:799–804. 10.1073/pnas.97.2.799 10639160 PMC15411

[84] Yu L Li R Liu W Zhou Y Li Y Qin Y. Protective effects of wheat peptides against ethanol-induced gastric mucosal lesions in rats: vasodilation and anti-inflammation. Nutrients. (2020) 12:2355. 10.3390/nu12082355 32784583 PMC7469019

[85] Lee D Song M Hong K Yun S Han Y Kim E. Low dose administration of mature silkworm powder induces gastric mucosal defense factors in ethanol-induced gastric injury rat model. Food Sci Biotechnol. (2023) 32:1551–9. 10.1007/s10068-023-01278-1 37637840 PMC10449703

[86] Beck PL Wong JF Li Y Swaminathan S Xavier RJ Devaney KL Podolsky DK. Chemotherapy-and radiotherapy-induced intestinal damage is regulated by intestinal trefoil factor. Gastroenterology. (2004) 126:796–808. 10.1053/j.gastro.2003.12.004 14988834

[87] Ethgen LM Pastore C Lin C Reed DR Hung L Douglas B. Trefoil factor 3-Lingo2 axis restrains proliferative expansion of type-1 T helper cells during GI nematode infection. Mucosal Immunol. (2024) 17:238–56. 10.1016/j.mucimm.2024.02.003 38336020 PMC11086637

[88] Aziz RS Siddiqua A Shahzad M Shabbir A Naseem N. Oxyresveratrol ameliorates ethanol-induced gastric ulcer via downregulation of IL-6, TNF-α, NF-κB, and COX-2 levels, and upregulation of TFF-2 levels. Biomed Pharmacother. (2019) 110:554–60. 10.1016/j.biopha.2018.12.002 30530291

[89] Zeng Y Wang Z Zou T Chen J Li G Zheng L. Bacteriophage as an alternative to antibiotics promotes growth performance by regulating intestinal inflammation, intestinal barrier function and gut microbiota in weaned piglets. Front Vet Sci. (2021) 8:623899. 10.3389/fvets.2021.623899 33585620 PMC7874526

[90] Chen H Mao X He J Yu B Huang Z Yu J. Dietary fibre affects intestinal mucosal barrier function and regulates intestinal bacteria in weaning piglets. Br J Nutr. (2013) 110:1837–48. 10.1017/S0007114513001293 23656640

[91] Wang LX Zhu F Li JZ Li YL Ding XQ Yin J. Epidermal growth factor promotes intestinal secretory cell differentiation in weaning piglets via wnt/β-catenin signalling. Animal. (2020) 14:790–8. 10.1017/S1751731119002581 31650938

[92] Ider M Yildiz R Naseri A Gülersoy E Alkan F Ok M. Investigation of gastrointestinal injury-related biomarkers in dairy cattle with displaced abomasum. Vet Med Sci. (2023) 9:2893–900. 10.1002/vms3.1292 37776262 PMC10650368

[93] Rowe A Gondro C Emery D Sangster N. Sequential microarray to identify timing of molecular responses to *Haemonchus contortus* infection in sheep. Vet Parasitol. (2009) 161:76–87. 10.1016/j.vetpar.2008.12.023 19200661

[94] Nagaraj SH Harsha HC Reverter A Colgrave ML Sharma R Andronicos N. Proteomic analysis of the abomasal mucosal response following infection by the nematode, *Haemonchus contortus*, in genetically resistant and susceptible sheep. J Proteomics. (2012) 75:2141–52. 10.1016/j.jprot.2012.01.016 22285630

[95] Menzies M Reverter A Andronicos N Hunt P Windon R Ingham A. Nematode challenge induces differential expression of oxidant, antioxidant and mucous genes down the longitudinal axis of the sheep gut. Parasite Immunol. (2010) 32:36–46. 10.1111/j.1365-3024.2009.01156.x 20042006

[96] Jing C Niu J Liu Y Jiao N Huang L Jiang S. Tannic acid extracted from galla chinensis supplementation in the diet improves intestinal development through suppressing inflammatory responses via blockage of NF-κB in broiler chickens. Animals. (2022) 12:2397. 10.3390/ani12182397 36139256 PMC9495145

[97] Zhu L Lu X Liu L Voglmeir J Zhong X Yu Q. *Akkermansia muciniphila* protects intestinal mucosa from damage caused by *S. pullorum* by initiating proliferation of intestinal epithelium. Vet Res. (2020) 51:1–9. 10.1186/s13567-020-00755-3 32138776 PMC7057645

[98] Orso C Cony BL Silva JP Furtado JCV Mann MB Frazzon J. Effect of live eimeria vaccination or salinomycin on growth and immune status in broiler chickens receiving in-feed inclusion of gelatin and vitamin E. Poult Sci. (2022) 101:102206. 10.1016/j.psj.2022.102206 36334427 PMC9627594

[99] Bijanzad P Momayez R Fard MHB Hablolvarid MH Mahmoodzadeh M Moghaddam ARJ. Study on clinical aspects of SPF chickens infected with H9N2 subtype of avian influenza virus. Ann Biol Res. (2013) 4:81–5.

[100] Xie Y Li J Liu D Wu B Zhao H Liu G. Dietary ethylenediamine dihydroiodide improves intestinal health in cherry valley ducks. Poult Sci. (2023) 102:103022. 10.1016/j.psj.2023.103022 37639753 PMC10477681

[101] Liu Y Wang Q Liu H Niu J Jiao N Huang L. Effects of dietary bopu powder supplementation on intestinal development and microbiota in broiler chickens. Front Microbiol. (2022) 13:1019130. 10.3389/fmicb.2022.1019130 36312926 PMC9612830

[102] Pender CM Kim S Potter TD Ritzi MM Young M Dalloul RA. In ovo supplementation of probiotics and its effects on performance and immune-related gene expression in broiler chicks. Poult Sci. (2017) 96:1052–62. 10.3382/ps/pew381 28158826

[103] Lin A Yan X Wang H Su Y Zhu W. Effects of lactic acid bacteria-fermented formula milk supplementation on ileal microbiota, transcriptomic profile, and mucosal immunity in weaned piglets. J Anim Sci Biotechnol. (2022) 13:113. 10.1186/s40104-022-00762-8 36199127 PMC9536082

[104] Deng Z Dai J Wei Y Ma Y Mao Y Zhang J. Comparison between *Lactobacillus rhamnosus* GG and LuxS-deficient strain in regulating gut barrier function and inflammation in early-weaned piglets. Front Immunol. (2022) 13:1080789. 10.3389/fimmu.2022.1080789 36569920 PMC9773554

[105] Limbach JR Espinosa CD Perez-Calvo E Stein HH. Effect of dietary crude protein level on growth performance, blood characteristics, and indicators of intestinal health in weanling pigs. J Anim Sci. (2021) 99:skab166. 10.1093/jas/skab166 34019637 PMC8202089

[106] Khoder G Al-Yassir F Al Menhali A Saseedharan P Sugathan S Tomasetto C. Probiotics upregulate trefoil factors and downregulate pepsinogen in the mouse stomach. Int J Mol Sci. (2019) 20:3901. 10.3390/ijms20163901 31405107 PMC6719917

[107] Tang Z Liu J Sun Z Li J Sun W Mao J. Protective effects of taurine on growth performance and intestinal epithelial barrier function in weaned piglets challenged without or with lipopolysaccharide. Anim Prod Sci. (2017) 58:2011–20. 10.1071/AN16249

[108] Chen YP Cheng YF Li XH Zhang H Yang WL Wen C. Dietary palygorskite supplementation improves immunity, oxidative status, intestinal integrity, and barrier function of broilers at early age. Anim Feed Sci Technol. (2016) 219:200–9. 10.1016/j.anifeedsci.2016.06.013

[109] Chang HM Loh TC Foo HL Lim ETC. *Lactiplantibacillus plantarum* postbiotics: alternative of antibiotic growth promoter to ameliorate gut health in broiler chickens. Front Vet Sci. (2022) 9:883324. 10.3389/fvets.2022.883324 35859810 PMC9289564

[110] Wu C Yang Z Song C Liang C Li H Chen W. Effects of dietary yeast nucleotides supplementation on intestinal barrier function, intestinal microbiota, and humoral immunity in specific pathogen-free chickens. Poult Sci. (2018) 97:3837–46. 10.3382/ps/pey268 29945221

[111] Leonard SG Sweeney T Bahar B Lynch BP O'Doherty JV. Effect of dietary seaweed extracts and fish oil supplementation in sows on performance, intestinal microflora, intestinal morphology, volatile fatty acid concentrations and immune status of weaned pigs. Br J Nutr. (2011) 105:549–60. 10.1017/S0007114510003739 20875191

[112] Khailova L Dvorak K Arganbright KM Halpern MD Kinouchi T Yajima M. Bifidobacterium bifidum improves intestinal integrity in a rat model of necrotizing enterocolitis. Am J Physiol Gastrointest Liver Physiology. (2009) 297:G940–9. 10.1152/ajpgi.00141.2009 20501441 PMC2777452

[113] Qi L Mao H Lu X Shi T Wang J. Corrigendum: cinnamaldehyde promotes the intestinal barrier functions and reshapes gut microbiome in early weaned rats. Front Nutr. (2022) 9:1038451. 10.3389/fnut.2022.1038451 36245511 PMC9562929

